# Hyaluronic-Acid-Coated Sterosome for Dasatinib Delivery in Hepatocellular Carcinoma: Preparation, Physicochemical Characterization, and In Vitro Evaluation

**DOI:** 10.3390/biomimetics10080552

**Published:** 2025-08-21

**Authors:** Chae Yeong Lee, Jeong Min Lee, Chung-Sung Lee, Hee Sook Hwang

**Affiliations:** 1 Department of Pharmaceutical Engineering, Dankook University, Cheonan 31116, Republic of Korea; 2 Department of Pharmaceutical Engineering, Soonchunhyang University, Asan 31538, Republic of Korea

**Keywords:** hepatocellular carcinoma, dasatinib, sterosome, hyaluronic acid, CD44 targeting

## Abstract

Hepatocellular carcinoma (HCC) is a leading cause of cancer-related death worldwide, and treatment remains challenging due to high recurrence rates, resistance to chemotherapy, and severe side effects. Dasatinib (Das) has shown therapeutic potential against HCC, but its clinical use is limited by poor bioavailability and short half-life (~3–4 h). Here, we developed a hyaluronic acid (HA)-coated sterosome for targeted and sustained delivery of Das to CD44-overexpressing HCC cells. Sterosomes composed of octadecylamine and cholesterol at a 5:5 (*v*/*v*) ratio were prepared via thin-film hydration and sonication, yielding stable particles (~90 nm) with high encapsulation efficiency (EE ~72%) for uncoated vesicles and ~58% after HA coating. HA-sterosomes (HA-St-Das) exhibited a uniform size (≈200 nm) and negative surface charge (–26 mV), with improved storage stability and resistance to lyophilization. In vitro release studies demonstrated pH-responsive Das release accelerated under acidic conditions (pH 6.0–5.0), mimicking tumor and lysosomal environments. In HepG2 cells, HA-St-Das exhibited enhanced cytotoxicity (IC50 ~7.0 μM) and prolonged intracellular retention compared to free Das and uncoated carriers. Fluorescence microscopy confirmed receptor-mediated uptake via CD44, leading to gradual and sustained intracellular delivery. Overall, the HA-St-Das system provides biocompatible, targeted, and controlled Das delivery, addressing key limitations of current liver cancer therapies and representing a promising nanomedicine platform for further development.

## 1. Introduction

Liver cancer is a major global health burden, ranking as the fourth leading cause of cancer-related deaths worldwide, and the second among men [1,2]. Among its subtypes, hepatocellular carcinoma (HCC) accounts for approximately 75–85% of all liver cancers. Despite the availability of clinical interventions such as surgical resection, liver transplantation, chemotherapy, radiotherapy, and molecular targeted therapy, the long-term prognosis of HCC remains poor due to high recurrence rates, metastasis, and resistance to conventional treatments [2,3,4,5]. These limitations underscore the urgent need for new and more effective therapeutic strategies.

To address these challenges, nanodrug delivery systems (NDDS) have emerged as promising platforms for enhancing drug efficacy and reducing systemic toxicity through tumor-targeted delivery [6]. Nanoparticles (NPs), including liposomes and micelles, have been widely investigated for their ability to encapsulate hydrophobic or hydrophilic drugs while improving pharmacokinetics and biodistribution [7,8,9,10]. However, conventional lipid-based nanocarriers often exhibit low encapsulation efficiency, limited structural stability, and premature drug leakage, especially for hydrophilic drugs like tyrosine kinase inhibitors [11,12,13,14].

Recently, a novel liposome-mimicking nanocarrier called the sterosome has attracted attention for its enhanced structural stability and encapsulation capacity. Unlike traditional phospholipid-based liposomes, sterosomes are composed of non-phospholipid surfactants and high sterol content, forming robust unilamellar vesicles with tunable size and improved drug retention [15,16,17,18]. Sterosomes exhibit prolonged circulation time, low production cost, and excellent storage stability, making them attractive candidates for clinical translation [13,14,19,20,21]. Moreover, they are highly adaptable for surface modification and payload functionalization, broadening their potential in targeted cancer therapy.

For the therapeutic payload, dasatinib (Das) was selected. Das is an FDA-approved potent tyrosine kinase inhibitor (TKI) known to inhibit Src family kinases (SFKs), a group of signaling molecules that promote tumor proliferation, migration, and metastasis in HCC [22,23,24,25]. Despite its therapeutic potential, Das suffers from rapid metabolism and a short plasma half-life (~3–4 h), which limit its clinical efficacy. Therefore, an effective and sustained delivery system is essential to realize its full anticancer potential.

To achieve liver cancer-specific targeting, hyaluronic acid (HA) is employed as a surface coating material for the sterosome. HA is a naturally occurring polysaccharide known for its excellent biodegradability, biocompatibility, and tumor-targeting ability via specific binding to CD44 receptors, which are overexpressed on the surface of HCC and other cancer cells [20,21,26,27]. In particular, CD44 variant isoforms such as CD44v6 are highly expressed in aggressive liver tumors and have been associated with poor prognosis [28]. Moreover, HA can also interact with hyaluronan-mediated motility receptor (RHAMM), further enhancing cancer cell selectivity [29,30]. Coating the sterosome with HA not only improves colloidal stability and circulation time but also enables ligand-mediated endocytosis into CD44-positive tumor cells, thereby enhancing intracellular drug accumulation and therapeutic efficacy [21,26,28,29,31,32].

**Figure 1:**
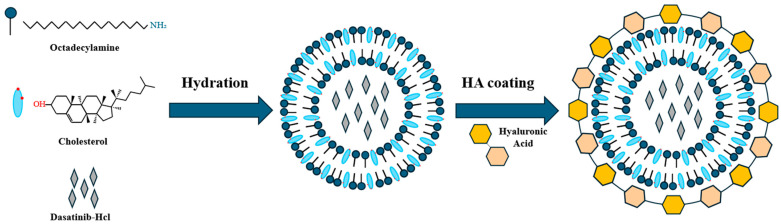
Schematic illustration of the preparation and surface modification of dasatinib-loaded sterosomes (HA-St-Das). Sterosomes were prepared using a thin-film hydration method with octadecylamine and cholesterol as the main lipid components and dasatinib hydrochloride (Das-) as the encapsulated drug. The resulting unilamellar vesicles encapsulate dasatinib within the hydrophobic bilayer. Subsequently, hyaluronic acid (HA) was coated onto the sterosome surface to improve colloidal stability, enhance biocompatibility, and enable active targeting of CD44-overexpressing hepatocellular carcinoma (HCC) cells.

In this study, we develop a hyaluronic acid-coated sterosome (HA-St) encapsulating Das (HA-St-Das) for targeted delivery to hepatocellular carcinoma cells (Figure 1). By systematically optimizing the composition of the sterosome and the HA coating conditions, we sought to construct a nanocarrier that exhibits favorable physicochemical properties, enhanced biocompatibility, and selective uptake by CD44-overexpressing liver cancer cells. Although HA-functionalized nanocarriers and sterosomes have been individually studied, to the best of our knowledge, this is the first study to combine HA-coated sterosomes for the targeted delivery of dasatinib to HCC cells. This novel formulation integrates the structural stability of sterosomes with HA-mediated CD44 targeting and pH-responsive release, offering a unique and effective nanoplatform for liver cancer therapy.

## 2. Materials and Methods

### 2.1. Materials

Octadecylamine (OA, ≥99%), Trizma^®^ base, and Hoechst 33342 were purchased from Sigma-Aldrich (St. Louis, MO, USA). Cholesterol (Chol, ≥95%,), Nile Red, and 3-(4,5-dimethylthiazol-2-yl)-2,5-diphenyltetrazolium bromide (MTT) were obtained from Tokyo Chemical Industry Co., Ltd. (Tokyo, Japan). Dasatinib hydrochloride (Das-HCl) was purchased from Macklin Biochemical Technology Co., Ltd. (Shanghai, China). Ethanol (EtOH, ≥99.99%) was obtained from Samchun Chemicals (Seoul, Republic of Korea). Sodium hyaluronate (weight-average molecular weight 1300 kDa, ≥98%) was purchased from Cosnet (Yecheon, Republic of Korea). Phosphate-buffered saline (PBS, 10×) was purchased from Dyne Bio (Seongnam, Republic of Korea). Tween^®^ 80 and dimethyl sulfoxide (DMSO) were purchased from HanLAB (Cheongju, Republic of Korea). Dulbecco’s Modified Eagle’s Medium (DMEM) and fetal bovine serum (FBS) were obtained from Welgene (Gyeongsan, Republic of Korea). Antibiotic–antimycotic solution (100×) was purchased from Cytiva (Amersham, UK).

### 2.2. Preparation of HA-Coated Das-Loaded Sterosomes (HA-St-Das)

Sterosomes were prepared using the thin-film hydration method [13,17,33]. Briefly, Chol and OA were mixed at the desired ratio and dissolved in ethanol in a round-bottom flask. The lipid solution was subjected to rotary evaporation at 60 °C under reduced pressure (approximately 150 mbar) to form a uniform, thin lipid film on the inner wall of the flask. Complete removal of the organic solvent was ensured by maintaining vacuum conditions. The resulting dry lipid film was then hydrated with TRIS buffer (0.2 M Tris, 1.37 M NaCl, pH 7.4), followed by sonication in a bath sonicator for 30 min to facilitate vesicle formation and homogenization.

HA-St-Das nanoparticles were prepared using the thin-film hydration method followed by surface modification with HA. To coat the surface with HA, St-Das, HA solution (1.0 mg/mL), and distilled water (DW) were mixed in a volume ratio of 2:1:1 and vortexed vigorously for 1 min to obtain HA-St-Das.

### 2.3. Characterization of St-Das and HA-St-Das

The particle diameter and zeta potential of the unmodified sterosome, dasatinib-loaded sterosome (St-Das), and hyaluronic acid-coated dasatinib-loaded sterosome (HA-St-Das) were measured using dynamic light scattering (DLS) with a Zetasizer Nano series instrument (Malvern Instruments, Worcestershire, UK). All measurements were performed at 25 °C, and data are reported as the mean ± standard deviation (SD) from three independent experiments. The morphologies of sterosomes were examined using transmission electron microscopy (TEM; JEM-2100F, JEOL Ltd., Tokyo, Japan).

The encapsulation efficiency (EE) of Das-HCl in St-Das and HA-St-Das was determined using UV–visible spectroscopy [34]. Briefly, 100 µL of the nanoparticle suspension was mixed with 400 µL of methanol and vortexed for 1 min to release the encapsulated drug. The Das concentration was quantified based on a calibration curve prepared in Tris-buffered saline (TBS)/methanol (5:2, *v*/*v*), exhibiting a correlation coefficient (*R*^2^) of 0.9958. The absorbance was measured at 325 nm using a microplate spectrophotometer (Synergy™ HTX, BioTek Instruments, Winooski, VT, USA). *EE* was calculated using the following equation:

$$EE\left(\%\right)=\frac{W_{\mathrm{Measurement}}}{W_{Control}}\times 100\%$$

where *W_Measurement_* represents the weight of Das from the extracted sample and *W_Control_* is the weight corresponding to the total amount of Das initially added.

The drug loading content (*DLC*) was also calculated to assess the relative amount of Das incorporated into the nanoparticle system. *DLC* was determined according to the following equation:

$$DLC\left(\%\right)=\frac{Total\;amount\;of\;dasatinib}{Total\;amount\;of\;sterosome}\times 100\%$$

### 2.4. Lyophilization of Sterosome

To evaluate the freeze-drying stability of the sterosome, 5% (*w*/*v*) sucrose was added as a cryoprotectant to the nanoparticle suspension and mixed thoroughly. The sample was then stored at −60 °C until completely frozen. Lyophilization was performed using a laboratory freeze dryer (TFD 8501, IlshinBioBase Co., Dongducheon, Republic of Korea) with the chamber temperature maintained at −80 °C and pressure kept below 0.1 mbar for 72 h under vacuum conditions. After lyophilization, the dried sterosome powder was reconstituted in distilled water (DW), and its particle size and zeta potential were measured using the DLS technique as described in Section 2.3.

### 2.5. Stability of Sterosome

The storage stability of St-Das and HA-St-Das was evaluated by monitoring changes in particle size over time under two different storage conditions. For aqueous-state formulations, each nanoparticle dispersion was stored at 4 °C in tightly sealed glass vials, and particle size was measured at defined intervals using DLS as described in Section 2.3. For lyophilized samples, stability was assessed after storage at −20 °C for one month, followed by reconstitution in DW prior to measurement. All measurements were conducted in triplicate, and results were expressed as the mean ± standard deviation (SD).

### 2.6. In Vitro Drug Release

The in vitro drug release profiles of Nile Red-loaded sterosomes (St-NR) and HA-coated sterosomes (HA-St-NR) were evaluated using the dialysis method under simulated physiological and pathological conditions. Phosphate-buffered saline (PBS, pH 7.4, 6.0, and 5.0) containing 0.2% (*w*/*v*) Tween^®^ 80 was used as the release medium to mimic the physiological environment (pH 7.4), tumor microenvironment (pH 6.0), and lysosomal conditions (pH 5.0). Briefly, 1 mL of St-NR or HA-St-NR suspension was transferred into a dialysis membrane bag (molecular weight cut-off: specify MWCO) and immersed in 20 mL of the corresponding release medium. The system was maintained at 37 °C (MCO-15AC, SANYO, Osaka, Japan) with continuous stirring at 200 rpm (MS2026, Mi-sung, Seoul, Republic of Korea). At predetermined time points for 240 h, the entire external medium was collected and replaced with an equal volume of fresh PBS to maintain sink conditions. The concentration of Nile Red released into the medium was determined using fluorescence spectrophotometry. A standard calibration curve was generated (correlation coefficient *R*^2^ = 0.9995) for quantification. The absorbance was measured at 555 nm using a microplate spectrophotometer (Synergy™ HTX, BioTek Instruments, Winooski, VT, USA).

### 2.7. Cell Culture and Incubation Conditions

The human hepatoblastoma cell line HepG2 was obtained from the Korean Cell Line Bank (Seoul, Republic of Korea). Cells were cultured in Dulbecco’s Modified Eagle’s Medium (DMEM) supplemented with 10% (*v*/*v*) fetal bovine serum (FBS) and 1% (*v*/*v*) penicillin–streptomycin solution (final concentration: 100 U/mL penicillin and 100 µg/mL streptomycin). Cultures were maintained in a humidified incubator at 37 °C with 5% CO_2_. Cells were subcultured every 2–3 days using standard trypsinization procedures and fresh complete medium.

### 2.8. Cell Cytotoxicity Assay

The cytotoxicity of Das, St-Das, and HA-St-Das was evaluated using the MTT assay. HepG2 cells were seeded in 96-well plates at a density of 5 × 10^3^ cells/well and incubated for 24 h at 37 °C in a humidified atmosphere containing 5% CO_2_. After incubation, 20 µL of drug-containing medium, prepared with various concentrations of Empty sterosome, HA-St, Das, St-Das, or HA-St-Das, was added directly to each well without removing the original medium. The cells were then further incubated for 24 h under the same conditions. Subsequently, 25 µL of MTT solution was added to each well, followed by a 4 h incubation to allow formazan crystal formation. The medium was then carefully removed and replaced with 200 µL of dimethyl sulfoxide (DMSO) to dissolve the formazan crystals. After 30 min of incubation at room temperature, the absorbance was measured at 595 nm using a microplate reader. *Cell viability* (%) was calculated using the following equation:

$$Cell\;viability\left(\%\right)=\frac{A_{Measurement}-A_{Blank}}{A_{Control}-A_{Blank}}\times 100\%$$

where *A_Measurement_* is the absorbance of the treated sample, *A_Control_* is the absorbance of untreated control cells, and *A_Blank_* is the absorbance of blank wells without cells.

The IC_50_ values of Das, St-Das, and HA-St-Das were determined from the MTT assay data to enable comparative evaluation of cytotoxicity among the different formulations.

### 2.9. Cellular Uptake

The cellular uptake of NR, St-NR, and HA-St-NR was evaluated in HepG2 cells using fluorescence microscopy. HepG2 cells were seeded in 6-well plates at a density of 3 × 10^4^ cells/well and cultured for 24 h at 37 °C in a humidified incubator containing 5% CO_2_. After incubation, the cells were washed with phosphate-buffered saline (PBS), and the medium was replaced with 1.8 mL of fresh culture medium containing 200 µL of NR, St-NR, or HA-St-NR. Following incubation with the nanoparticles for 2, 4, 6, 8, and 12 h, the cells were washed three times with PBS to remove uninternalized particles. The cell nuclei were then stained with Hoechst 33342 for fluorescent visualization. Fluorescence images were captured at 2, 4, 8, and 12 h using a fluorescence microscope to observe the intracellular distribution of NR-loaded formulations. Quantitative analysis of intracellular fluorescence intensity was performed using ImageJ software (1.54p). Time-dependent uptake behavior was further analyzed by generating kinetic graphs based on fluorescence intensity values obtained at each time point.

### 2.10. Statistical Analysis

All experiments were performed in triplicate, and the results are presented as the mean ± standard deviation (SD). Statistical analysis was conducted using one-way analysis of variance (ANOVA) followed by Tukey’s post hoc test for multiple comparisons. A *p*-value of less than 0.05 was considered statistically significant.

## 3. Results and Discussion

### 3.1. Preparation of Sterosomes

**Figure 2:**
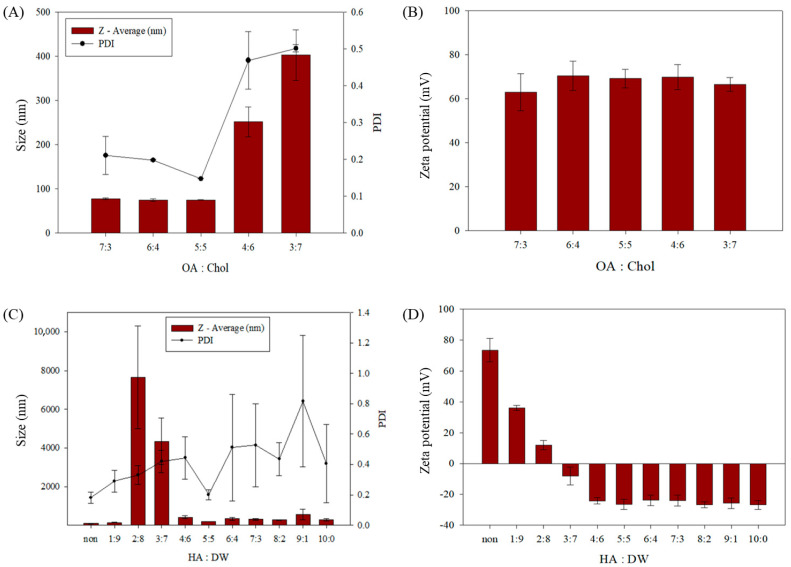
Characterization of sterosomes prepared with various OA:Chol ratios. (**A**) Particle size and polydispersity index (PDI); (**B**) zeta potential values. Characterization of HA-St-Das nanoparticles prepared with varying ratios of 1.0 mg/mL HA and distilled water (DW). (**C**) Particle size and polydispersity index (PDI). (**D**) Zeta potential. Data represent the mean ± standard deviation (SD) of three independent experiments (*n* = 3).

To identify the optimal formulation for sterosome preparation, various molar ratios of OA and Chol were evaluated using the thin-film hydration method. Sterosomes were prepared at OA:Chol ratios of 7:3, 6:4, 5:5, 4:6, and 3:7 (*v*/*v*), and their physicochemical properties—including particle size, zeta potential, and polydispersity index (PDI)—were analyzed using DLS (Figure 2 and Table 1). Among the tested formulations, the 5:5 OA:Chol ratio yielded in the smallest particle size and lowest PDI, indicating the formation of uniform and stable nanoparticles. In contrast, formulations containing higher Chol proportions (4:6 and 3:7) exhibited increased particle sizes and broader size distributions, suggesting compromised colloidal stability. Based on these observations, the 5:5 ratio was selected as the optimal formulation for subsequent experiments. Notably, the 5:5 OA:Chol composition corresponds to a relatively high cholesterol content compared to conventional lipid-based nanocarriers. In typical liposomal drug delivery systems, cholesterol is incorporated at 20–30 mol% relative to phospholipids to modulate membrane fluidity and reduce leakage, with formulations rarely exceeding 40 mol% [35,36]. In contrast, the sterosome system utilized in this study contains 50 mol% cholesterol, enhancing structural rigidity and storage stability. This high Chol content is a defining feature of sterosomes and plays a crucial role in maintaining vesicle integrity and drug retention under physiological conditions.

To improve the biocompatibility and cancer-targeting ability of the sterosome, surface modification with HA was performed. HA was selected as a functional coating agent due to its high affinity for CD44 receptors, which are overexpressed in many cancer cells, including hepatocellular carcinoma. To determine the optimal HA coating conditions, various volume ratios of St-Das, a 1.0 mg/mL HA solution, and DW were tested. The resulting HA-coated nanoparticles (HA-St-Das) were characterized in terms of particle size, zeta potential, and polydispersity index (PDI) (Figure 2C,D and Table 2). Maintaining a stable particle size and an appropriately negative zeta potential was critical for ensuring colloidal stability and systemic circulation potential. Inadequate HA coating resulted in poor surface coverage and particle agglomeration due to electrostatic instability, while excessive HA content led to the formation of a thick polymer layer, which increased particle size unnecessarily [37]. A thick HA layer can also hinder cellular uptake by limiting interactions between the nanoparticle surface and the cell membrane [38].

To further enhance coating stability and functionality, high molecular weight HA (1300 kDa) was used in this study. High molecular weight HA is known to form compact and less hydrated surface films, which improve coating integrity and structural stability during circulation [39]. After evaluating several HA:DW ratios, a 2:1:1 (St-Das:HA:DW) volume ratio was identified as optimal. Under these conditions, the HA-St-Das nanoparticles exhibited a uniform particle size of approximately 203 ± 1.2 nm and a zeta potential of −26.41 ± 3.24 mV, indicating good dispersion stability and biocompatibility. These results suggest that the selected HA coating condition balances the need for colloidal stability and cellular uptake efficiency, making the HA-St-Das formulation suitable for systemic administration and tumor targeting.

### 3.2. Characterization of the Sterosome

**Figure 3:**
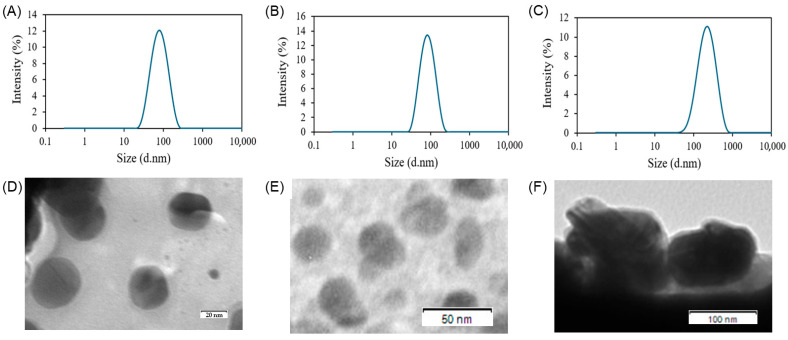
Dynamic light scattering (DLS) profiles and transmission electron microscopy (TEM) images of (**A**) empty sterosome, (**B**) St-Das, and (**C**) HA-St-Das. The size distribution and polydispersity index (PDI) were determined by DLS. Representative TEM images of (**D**) empty sterosome, (**E**) St-Das, and (**F**) HA-St-Das present the morphology and relative size of the nanoparticles. Scale bars in TEM images represent (**D**) 20, (**E**) 50, and (**F**) 100 nm. Data are presented as mean ± SD from three independent experiments (*n* = 3).

The physicochemical properties of the uncoated and HA-coated sterosome formulations were evaluated by measuring particle size, zeta potential, PDI, encapsulation efficiency, and drug loading content. DLS analysis revealed that the drug-unloaded empty sterosome exhibited a particle size of 75.1 ± 0.7 nm and a zeta potential of 69.0 ± 4.2 mV (Figure 3A and Table 3). Upon Das loading, the resulting St-Das particles had a slightly increased size of 89.1 ± 6.2 nm and a zeta potential of 73.6 ± 7.6 mV (Figure 3B). The PDI value for St-Das was 0.18 ± 0.04, indicating a monodisperse and uniform particle distribution. After surface modification with HA, the HA-St-Das formulation showed a significantly larger particle size of 203.0 ± 1.2 nm and a markedly reduced zeta potential of −26.4 ± 3.2 mV (Figure 3C), confirming successful coating with the anionic polysaccharide, HA. The PDI remained low at 0.20 ± 0.03, suggesting that the HA coating process did not adversely affect size uniformity. As shown in TEM images (Figure 3D–F), the sterosomes exhibited spherical nanostructures with 3D architectures, measuring under 100 nm in diameter before HA coating (empty sterosome and St-Das) and approximately 200 nm after HA coating (HA-St-Das), consistent with the DLS results.

EE of dasatinib was 71.87 ± 1.37% for St-Das and 57.78 ± 0.21% for HA-St-Das (Table 3). The reduced EE after HA coating may result from partial leakage or interference of HA with drug entrapment during the coating process [40]. DLC was also slightly decreased from 10.08 ± 0.18% in St-Das to 7.48 ± 0.03% in HA-St-Das. This reduction may have resulted from the increased total nanoparticle mass after HA addition and possible steric hindrance or molecular interactions that affect drug loading capacity [41].

**Figure 4:**
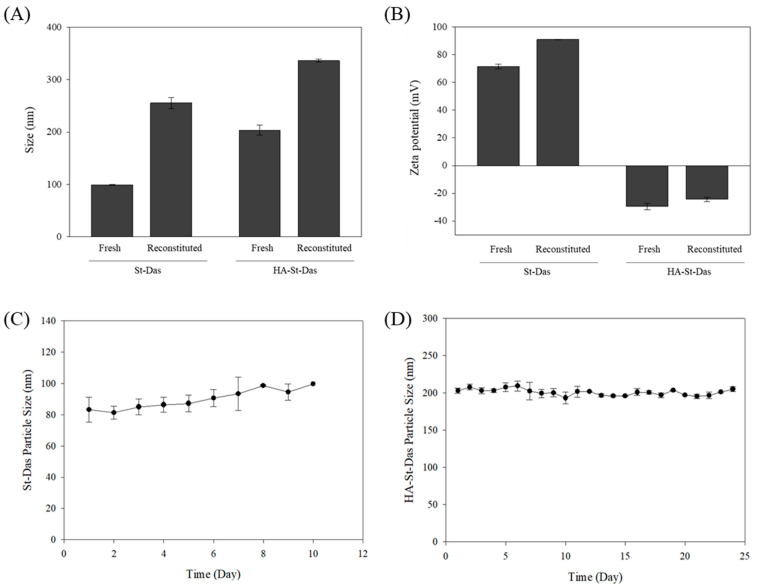
(**A**) Comparison of particle size and (**B**) zeta potential before and after lyophilization for St-Das and HA-St-Das. Lyophilized samples were stored at −20 °C for one month and reconstituted in distilled water (DW) prior to measurement. In vitro stability of (**C**) St-Das and (**D**) HA-St-Das stored at 4 °C. Particle size was measured using DLS over a 24-day period. Data are presented as mean ± SD (*n* = 3).

To investigate long-term storage stability, both St-Das and HA-St-Das were freeze-dried in the presence of 5% (*w*/*v*) sucrose as a cryoprotectant. The lyophilized samples were stored at −20 °C for one month and then reconstituted in distilled water for analysis. After reconstitution, both formulations exhibited increased particle sizes but retained their nanoscale characteristics. The reconstituted particle sizes were 255.4 ± 10.2 nm for St-Das and 336.3 ± 2.6 nm for HA-St-Das, while zeta potentials were 90.9 ± 0.2 mV and −24.4 ± 1.5 mV, respectively (Figure 4A,B). These results confirm that both formulations maintained colloidal integrity after one month in frozen storage and subsequent lyophilization.

The colloidal stability of the formulations was further assessed by storing St-Das and HA-St-Das at 4 °C and monitoring particle size over time. While St-Das exhibited a significant size increase after 10 days, HA-St-Das maintained a stable particle size for up to 24 days (Figure 4C,D). This indicates that HA coating substantially improves colloidal stability during refrigerated storage.

In addition to the lyophilization results shown in Figure 4A,B, where samples were stored at −20 °C for one month prior to reconstitution, these findings suggest that the HA-St-Das formulation maintains structural stability in both lyophilized and aqueous forms. Even without lyophilization, the liquid formulation of HA-St-Das remained sufficiently stable for short- to mid-term storage under standard laboratory conditions. In future studies, further optimization of lyophilized formulations, including the selection of appropriate cryoprotectants and freeze-drying conditions, will be investigated to enhance long-term shelf stability and facilitate clinical translation of this nanocarrier system.

### 3.3. In Vitro Drug Release

PBS solutions with different pH values (pH 7.4, 6.0, and 5.0) were used to simulate the normal physiological, tumor, and lysosomal environments, respectively, to evaluate whether the drugs encapsulated within the sterosome were effectively released at the tumor site. To confirm the release of drugs from within the sterosome, Nile Red-loaded sterosome was used in this study. Generally, dasatinib does not emit fluorescence signals; however, Nile Red is a fat-soluble fluorescent dye that emits a strong fluorescence signal when combined with intracellular lipids [14,42]. Additionally, it binds well to intracellular lipids, with its fluorescence spectrum varying depending on the lipid environment, enabling visual observation or quantification of the intracellular drug delivery process. Therefore, we conducted an in vitro assay using Nile Red instead of dasatinib [43].

**Figure 5:**
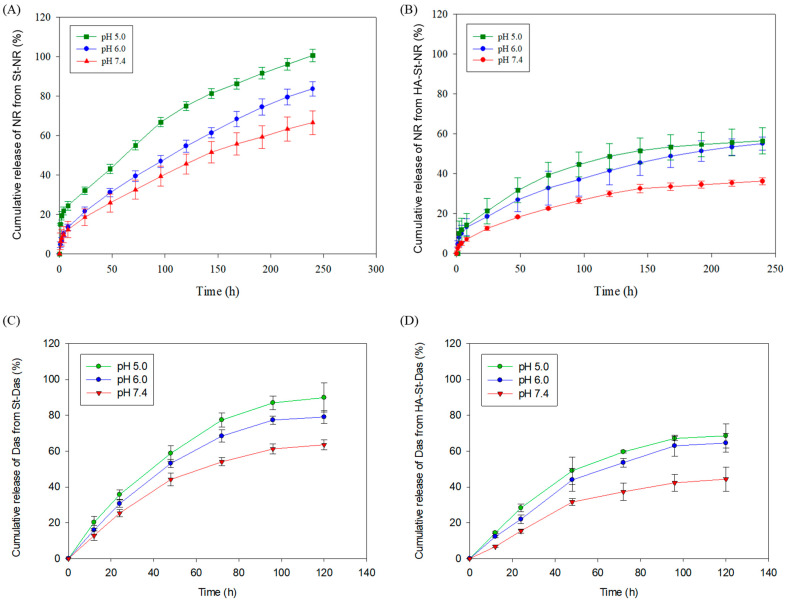
In vitro drug release profiles of the (**A**) St-NR, (**B**) HA-St-NR, (**C**) St-Das, and (**D**) HA-St-Das. Measurements were obtained from three independent experiments (*n* = 3). Data in graphs are expressed as mean ± SD.

Figure 5A shows the release rates of Nile Red from sterosome in PBS solution at pH 7.4, 6.0, and 5.0. After incubation for 240 h at pH 7.4, approximately 66% of Nile Red was released from the sterosome. At pH 6.0 and 5.0, Nile Red was released at approximately 83% and 100%, respectively. The release of Nile Red from HA-St-NR followed the same pattern (Figure 5B). After incubation for 240 h, cumulative release of Nile Red from HA-St-NR was 36% (pH 7.4), 54% (pH 6.0), and 56% (pH 5.0). These results indicate that the sterosome remains stable in the physiological environment and achieves targeted release in the tumor environment, thereby enhancing drug delivery to the target site and improving the therapeutic effect.

In addition, in vitro drug release studies were also conducted using Das-loaded sterosomes (St-Das and HA-St-Das) under the same pH conditions (Figure 5C,D). The release profiles of Das showed a similar pH-responsive trend to that observed with Nile Red; however, the cumulative amount of Das released over 5 days was comparable to the 10-day release of Nile Red. This relatively faster release of Das is likely due to its higher aqueous solubility, which facilitates diffusion from the sterosome matrix. These findings further confirm that the sterosome system can provide sustained release of the actual therapeutic drug while maintaining pH-responsive behavior.

### 3.4. In Vitro Cytotoxicity of Sterosome

**Figure 6:**
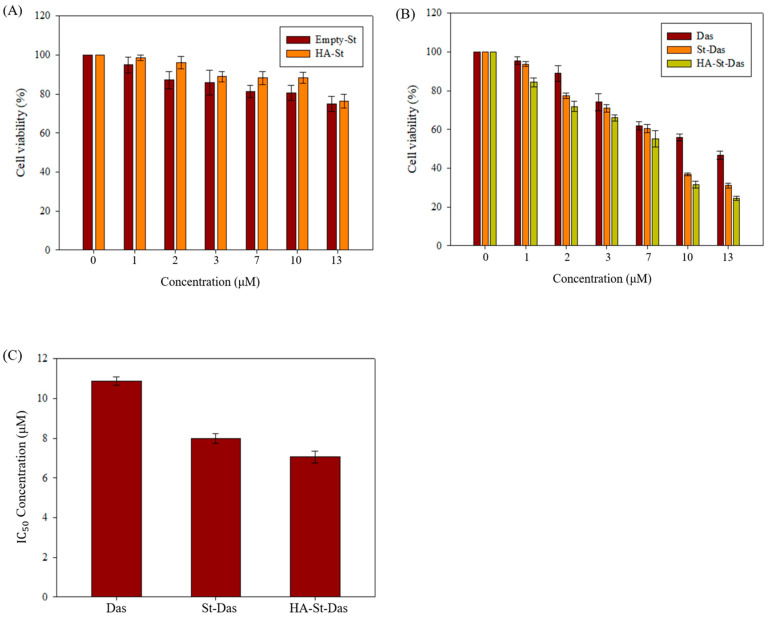
In vitro cytotoxicity of HepG2 cells treated with (**A**) drug-free empty sterosome and HA-St and (**B**) dasatinib (Das), St-Das, and HA-St-Das at various concentrations for 24 h. Cell viability was assessed using the MTT assay. (**C**) IC_50_ values of Das, St-Das, and HA-St-Das in HepG2 cells, determined from MTT assay data. Data are presented as mean ± SD (*n* = 3).

The cytotoxicity of sterosome carriers and the therapeutic efficacy of dasatinib-loaded formulations were evaluated using the MTT assay in HepG2 cells. First, to assess the biocompatibility of the nanocarriers, cells were treated with various concentrations of drug-free sterosome and HA-coated sterosome for 24 h. As shown in Figure 6A, both treatments resulted in cell viabilities exceeding 70%, indicating that the sterosome formulations themselves have low intrinsic cytotoxicity and are suitable for biomedical applications.

Next, the cytotoxic effects of free Das, St-Das, and HA-St-Das were examined. As presented in Figure 6B, all formulations demonstrated dose-dependent cytotoxicity after 24 h of incubation. Notably, St-Das and HA-St-Das induced significantly greater reductions in cell viability compared to free Das at equivalent concentrations. At a concentration of 13 μM, cell viability was 46.60 ± 2.09% for free Das, 30.97 ± 1.17% for St-Das, and 24.26 ± 1.04% for HA-St-Das. These results confirm that dasatinib delivered via sterosome enhances its anticancer activity and that the HA coating further augments this effect through improved cellular uptake and tumor targeting.

To further quantify the comparative efficacy of the formulations, IC_50_ values were calculated from the MTT assay results (Figure 6C and Table 4). The IC_50_ of free Das was 10.87 ± 0.22 μM, whereas St-Das and HA-St-Das showed significantly lower IC_50_ values of 7.99 ± 0.25 μM and 7.05 ± 0.29 μM, respectively. These data indicate that sterosome-based delivery improves the potency of Das and that HA surface modification further enhances this effect by promoting targeted uptake in CD44-overexpressing HCC cells (HepG2).

### 3.5. Cellular Uptake of Sterosome

**Figure 7:**
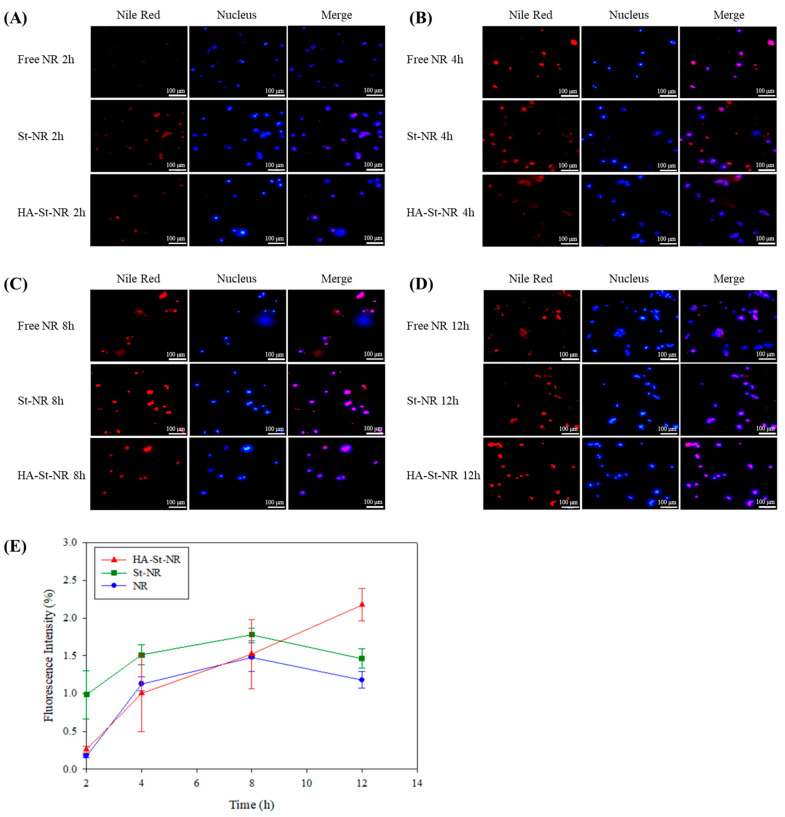
Fluorescence images confirming the uptake of free Nile Red (NR), St-NR, and HA-St-NR in HepG2 cells at (**A**) 2 h, (**B**) 4 h, (**C**) 8 h, and (**D**) 12 h. Scale bar: 100 µm. Red fluorescence indicates Nile Red in the sterosomes (Nile Red was used as a model drug encapsulated within the sterosome); blue fluorescence indicates Hoechst 33342 nuclear staining. (**E**) Quantitative fluorescence intensity profiles of NR, St-NR, and HA-St-NR in HepG2 cells at (**A**) 2 h, (**B**) 4 h, (**C**) 8 h, and (**D**) 12 h. Fluorescence intensity was quantified using ImageJ software from the microscopic images, and data represent the mean ± SD of three independent experiments (*n* = 3).

The cellular uptake of Nile Red (NR), NR-loaded sterosome (St-NR), and hyaluronic acid-coated St-NR (HA-St-NR) was evaluated in HepG2 cells using fluorescence microscopy. The particle size, zeta potential, PDI, EE, and DLC of St-NR and HA-St-NR did not differ significantly from those of their dasatinib-loaded sterosomes (St-Das and HA-St-Das) (Table 5). Cells were incubated with each formulation for 2, 4, 8, and 12 h, and intracellular fluorescence was observed to assess time-dependent nanoparticle internalization (Figure 7). At 2 h, free NR exhibited weak red fluorescence, indicating limited cellular uptake (Figure 7A). In contrast, St-NR showed significantly higher fluorescence intensity, suggesting efficient internalization facilitated by its cationic surface charge, which promotes electrostatic interactions with the negatively charged cell membrane. HA-St-NR also demonstrated visible fluorescence, albeit lower than that of St-NR, likely due to its anionic surface charge reducing nonspecific electrostatic interaction at early time points. At 4 h, free NR showed a marked increase in intracellular fluorescence, indicating a burst uptake phase (Figure 7B). Both St-NR and HA-St-NR also exhibited increased fluorescence compared to 2 h, with St-NR maintaining the highest signal intensity at this stage. After 8 h of incubation, the fluorescence signal of free NR plateaued, showing minimal increase relative to 4 h, suggesting rapid saturation and limited retention (Figure 7C). In contrast, both St-NR and HA-St-NR continued to exhibit increased fluorescence intensity, indicating sustained uptake over time. By 12 h, HA-St-NR surpassed St-NR in fluorescence intensity, demonstrating prolonged and cumulative intracellular accumulation (Figure 7D). This result suggests that HA-mediated, receptor-dependent endocytosis, combined with sterosome stability, enhances intracellular delivery and retention over extended periods. Overall, sterosome-based formulations (St-NR and HA-St-NR) exhibited a more gradual and sustained uptake pattern, in contrast to free NR, which showed rapid but short-lived uptake. Notably, HA-St-NR provided the most prolonged and regulated intracellular accumulation, supporting its potential as a targeted and efficient nanocarrier for liver cancer therapy.

Figure 7E presents the time-dependent intracellular uptake profiles of NR, St-NR, and HA-St-NR in HepG2 cells, as quantified by fluorescence intensity. These data are derived from the fluorescence images shown in Figure 7A–D. Although the absolute nanoparticle count was not directly measured, fluorescence intensity was used as a proxy for cellular uptake based on established literature, which supports a strong correlation between signal intensity and nanoparticle internalization [44,45]. Free NR displayed low fluorescence at 2 h but showed a rapid increase by 4 h, followed by a plateau at later time points (8 and 12 h). This pattern suggests a rapid but nonspecific uptake, with limited intracellular retention. In contrast, St-NR exhibited higher fluorescence intensity from the early time point (2 h) and continued to increase until 8 h, indicating efficient but eventually saturable uptake likely driven by electrostatic interactions between the cationic surface of the sterosome and the negatively charged cell membrane. After 8 h, no significant increase in fluorescence was observed, suggesting uptake saturation. HA-St-NR showed a markedly different profile. Although its initial uptake was lower than St-NR, likely due to its anionic surface, the fluorescence intensity increased steadily and significantly over 12 h, ultimately surpassing both NR and St-NR. This sustained uptake trend is indicative of receptor-mediated internalization via CD44, facilitated by the HA coating, as well as improved colloidal stability that prevents premature aggregation or clearance. Overall, the kinetic data clearly demonstrate that HA-St-NR achieves gradual, prolonged, and targeted intracellular accumulation, unlike free NR, which shows burst-like uptake, or St-NR, which reaches saturation prematurely. These results highlight the advantages of HA-coated sterosomes in regulating drug delivery kinetics and enhancing selective tumor targeting, thereby underscoring their potential utility for liver cancer therapy.

Although in vitro results demonstrated CD44-mediated uptake and pH-triggered release, further validation through in vivo biodistribution, pharmacokinetic, and therapeutic efficacy studies is necessary to confirm tumor-specific targeting and assess the translational potential of HA-St-Das. In addition, although the cytotoxic effects observed in HepG2 cells are consistent with the known mechanisms of dasatinib-induced apoptosis, additional mechanistic studies, such as CD44 knockdown, immunoassays for CD44 expression and downstream signaling, apoptosis assays, and cell cycle analysis, are needed to clarify the specific uptake and intracellular action mechanisms of HA-St-Das [46]. Since dasatinib is used in its unmodified form, its intracellular activity is expected to follow established pathways; however, mechanistic confirmation will help distinguish the contribution of the HA-coated sterosome as a targeted delivery system.

In agreement with previous studies, HA coating markedly enhanced colloidal stability and cellular uptake through CD44-mediated interactions. Arpicco et al. demonstrated that HA-modified liposomes improved nanoparticle dispersion stability and increased cellular internalization in CD44-overexpressing tumor cells, resulting in superior anticancer efficacy compared to uncoated counterparts [47]. Similarly, Park et al. reported that HA derivative (HA-ceramide)-coated nanoparticles showed prolonged circulation and enhanced tumor accumulation in vivo, supporting the role of HA as an effective targeting ligand [48]. Our findings are consistent with these reports, confirming that HA surface modification of sterosomes improves stability, facilitates targeted uptake, and enhances in vitro anticancer activity, thereby underscoring its potential for further translational development.

## 4. Conclusions

In this study, we successfully developed a HA-coated sterosome as a nanocarrier platform for the targeted delivery of dasatinib, demonstrating enhanced cellular uptake, stability, and cytotoxicity in HepG2 cells. The sterosome, formulated with a 5:5 ratio of OA and Chol, had a notably higher sterol content than conventional liposomal carriers. It exhibited superior structural stability, nanoscale size distribution (~90 nm for uncoated vesicles and ~200 nm after HA coating), and a positive surface charge favorable for drug encapsulation (EE% ~72% for St-Das and ~58% for HA-St-Das) and cellular interaction. HA functionalization significantly enhanced the biocompatibility, serum stability, and liver-cancer-targeting capability of the sterosome via CD44 receptor-mediated endocytosis. Physicochemical characterization confirmed that the HA coating modulated surface charge, maintained uniform particle size, and extended colloidal stability. Furthermore, HA-St-Das showed sustained pH-responsive drug release under tumor-relevant acidic conditions (accelerated at pH 6.0–5.0) and improved lyophilization stability, ensuring storage compatibility for potential clinical applications. Cellular assays using HepG2 cells validated the therapeutic potential of the system. MTT analysis demonstrated that HA-St-Das exerted significantly greater cytotoxicity than free Das or uncoated sterosome, with lower IC_50_ values (~7.0 μM). Time-resolved fluorescence microscopy revealed that HA-St-Das enabled gradual and prolonged intracellular accumulation, surpassing both free NR and St-NR formulations, thereby supporting enhanced tumor-targeted uptake and intracellular retention. This work not only presents a novel application of HA-coated sterosomes for Das delivery, but also highlights a strategically designed system that addresses multiple limitations of existing delivery strategies, including drug instability, rapid clearance, and nonspecific cytotoxicity. By integrating HA-mediated active targeting with the sterosome’s structural advantages, this platform offers a distinct innovation in the development of liver cancer therapeutics, addressing key challenges of conventional HCC therapies and providing a strategic foundation for advanced nanomedicine solutions.

## Figures and Tables

**Table 1:** Physicochemical characterization of sterosomes prepared with different OA:Chol ratios. Data include particle size, zeta potential, and polydispersity index (PDI). Values are presented as mean ± SD (*n* = 3).

OA:Cholesterol	Size (nm)	Zeta Potential (mV)	PDI
7:3	78.2 ± 1.9	63.0 ± 8.4	0.21 ± 0.05
6:4	74.8 ± 3.0	70.5 ± 6.6	0.20 ± 0.01
5:5	75.1 ± 0.7	69.2 ± 4.2	0.15 ± 0.01
4:6	251.9 ± 34.2	69.9 ± 5.7	0.47 ± 0.08
3:7	402.9 ± 57.7	66.5 ± 3.1	0.50 ± 0.01

**Table 2:** Physicochemical characteristics of HA-St-Das nanoparticles prepared with different ratios of 1.0 mg/mL HA and DW. Particle size, zeta potential, and polydispersity index (PDI) values are presented as mean ± SD (*n* = 3).

HA:DW (*v*/*v*)	Size (nm)	Zeta Potential (mV)	PDI
non	89.1 ± 6.2	73.6 ± 7.6	0.18 ± 0.04
1:9	137.5 ± 15.4	36.0 ± 1.6	0.29 ± 0.07
2:8	7660.3 ± 2642.3	11.9 ± 3.0	0.33 ± 0.06
3:7	4346.7 ± 1197.3	−8.0 ± 5.7	0.42 ± 0.07
4:6	409.9 ± 72.6	−24.2 ± 2.1	0.44 ± 0.14
5:5	203.4 ± 1.2	−26.4 ± 3.2	0.20 ± 0.03
6:4	333.7 ± 71.8	−23.8 ± 3.4	0.51 ± 0.35
7:3	299.9 ± 41.4	−24.0 ± 3.5	0.53 ± 0.27
8:2	276.3 ± 9.4	−26.7 ± 1.9	0.44 ± 0.11
9:1	563.4 ± 274.1	−25.8 ± 3.4	0.82 ± 0.43
10:0	284.7 ± 69.6	−26.8 ± 2.9	0.41 ± 0.26

**Table 3:** Particle size, zeta potential, polydispersity index (PDI), encapsulation efficiency (EE, %), and drug loading content (DLC, %) of empty sterosome, St-Das, and HA-St-Das. Values are expressed as mean ± SD (*n* = 3).

	Size (nm)	Zeta Potential (mV)	PDI	EE (%)	DLC (%)
Empty sterosome	75.1 ± 0.7	69.0 ± 4.2	0.15 ± 0.01	-	-
St-Das	89.1 ± 6.2	73.6 ± 7.6	0.18 ± 0.01	77.81 ± 1.37	10.08 ± 0.18
HA-St-Das	203.0 ± 1.2	−26.4 ± 3.2	0.20 ± 0.03	57.78 ± 0.21	7.48 ± 0.03

**Table 4:** Half-maximal inhibitory concentration (IC_50_) values of Das, St-Das, and HA-St-Das in HepG2 cells. Values are expressed as mean ± SD (*n* = 3).

	Das	St-Das	HA-St-Das
IC_50_ (μM)	10.87 ± 0.22	7.99 ± 0.25	7.05 ± 0.29

**Table 5:** Particle size, zeta potential, polydispersity index (PDI), encapsulation efficiency (EE, %), and drug loading content (DLC, %) of St-NR and HA-St-NR. Values are expressed as mean ± SD (*n* = 3).

	Size (nm)	Zeta Potential (mV)	PDI	EE (%)	DLC (%)
St-NR	96.2 ± 9.8	61.7 ± 6.9	0.17 ± 0.03	62.96 ± 3.65	8.15 ± 0.47
HA-St-NR	178.4 ± 10.1	−34.1 ± 5.5	0.19 ± 0.04	51.21 ± 5.33	6.63 ± 0.69

## Data Availability

Dataset available on request from the authors.
